# Transcriptome Sequencing from Diverse Human Populations Reveals Differentiated Regulatory Architecture

**DOI:** 10.1371/journal.pgen.1004549

**Published:** 2014-08-14

**Authors:** Alicia R. Martin, Helio A. Costa, Tuuli Lappalainen, Brenna M. Henn, Jeffrey M. Kidd, Muh-Ching Yee, Fabian Grubert, Howard M. Cann, Michael Snyder, Stephen B. Montgomery, Carlos D. Bustamante

**Affiliations:** 1 Stanford University School of Medicine, Department of Genetics, Stanford, California, United States of America; 2 Stony Brook University, SUNY, Department of Ecology and Evolution, Stony Brook, New York, United States of America; 3 University of Michigan School of Medicine, Department of Human Genetics, Ann Arbor, Michigan, United States of America; 4 Foundation Jean Dausset, Centre d'Etude du Polymorphisme Humain, Paris, France; 5 Stanford University School of Medicine, Department of Pathology, Stanford, California, United States of America; Georgia Institute of Technology, United States of America

## Abstract

Large-scale sequencing efforts have documented extensive genetic variation within the human genome. However, our understanding of the origins, global distribution, and functional consequences of this variation is far from complete. While regulatory variation influencing gene expression has been studied within a handful of populations, the breadth of transcriptome differences across diverse human populations has not been systematically analyzed. To better understand the spectrum of gene expression variation, alternative splicing, and the population genetics of regulatory variation in humans, we have sequenced the genomes, exomes, and transcriptomes of EBV transformed lymphoblastoid cell lines derived from 45 individuals in the Human Genome Diversity Panel (HGDP). The populations sampled span the geographic breadth of human migration history and include Namibian San, Mbuti Pygmies of the Democratic Republic of Congo, Algerian Mozabites, Pathan of Pakistan, Cambodians of East Asia, Yakut of Siberia, and Mayans of Mexico. We discover that approximately 25.0% of the variation in gene expression found amongst individuals can be attributed to population differences. However, we find few genes that are systematically differentially expressed among populations. Of this population-specific variation, 75.5% is due to expression rather than splicing variability, and we find few genes with strong evidence for differential splicing across populations. Allelic expression analyses indicate that previously mapped common regulatory variants identified in eight populations from the International Haplotype Map Phase 3 project have similar effects in our seven sampled HGDP populations, suggesting that the cellular effects of common variants are shared across diverse populations. Together, these results provide a resource for studies analyzing functional differences across populations by estimating the degree of shared gene expression, alternative splicing, and regulatory genetics across populations from the broadest points of human migration history yet sampled.

## Author Summary

Previous gene expression studies have identified factors influencing population-level variation in gene regulation. However, these efforts have been limited to a small set of well-studied populations. By leveraging the high resolution of RNA sequencing and broad population sampling, we survey the landscape of transcriptome variation across a globally distributed set of seven populations that span a breadth of human genetic variation and major dispersal events. We assess differences in gene expression, transcript structure, and regulatory variation. We find only 44 transcripts that show significant differences in expression, likely as a result of the small sample size, but we find that 25% of the variance in gene expression is due to population differences. This is a larger fraction than previously observed, and it is likely due to the greater breadth of human diversity assayed in this study. We also find that population-specific variance is mostly due to transcription variability rather than the configuration of expressed gene products. Additionally, known common regulatory variants have similar effects across populations including those we study here. These data and results serve as a resource cataloging the wide array of gene expression regulation affecting population variation among diverse groups, improving our understanding of transcriptional diversity.

## Introduction

A central challenge in modern medical and population genomics is identifying trait-disposing genetic variants, interpreting their molecular consequences, and determining the transferability of their functional roles across individuals and populations. While genome-wide association studies (GWAS) have correlated an abundance of common and (increasingly) rare variants with disease, far fewer studies have pinpointed causal variants, discovered the biological mechanism of the association, or replicated their findings in different populations. Here, we build upon previous work using transcript abundance and splicing as model systems for understanding how population substructure can impact the genetic architecture of biomedical traits [1]–[4]. In particular, we focus on a set of populations that span the “Out-of-Africa” migration of anatomically modern humans using CEPH Human Genome Diversity Panel cell lines, for which we have collected an extensive ‘omics profile described below.

Genetic studies of microsatellites panels and single nucleotide polymorphisms (SNPs) have shown a decrease in genetic diversity as a function of a population's geographic distance from eastern or southern Africa [5]–[7]. This pattern fits a serial founder effect model, but it remains unclear whether transcriptome variation follows this pattern and how closely genetic effects on regulation mirror human migration history. Previous work has shown that population bottlenecks reduce heterozygosity and are associated with an accumulation of damaging and loss-of-function variation which can impact gene expression [8], [9]. However, further molecular work is needed to settle the controversy regarding demography and its impacts on the distribution of functional genetic variation among populations.

Gene expression studies within and between well-studied populations have been transformative in cataloging gene expression differences, expression quantitative trait loci (eQTLs) with different types of regulatory variants, as well as allele-specific expression (ASE) that underlie many disease associations [3], [10]–[16]. Technological advances in RNA sequencing and transcript assembly have also enabled analysis of variation in transcript structure and regulation of alternative splicing. For example, splicing ratios can differ between distant populations even in the absence of expression differences, and some population-specific splicing differences are involved in known disease-susceptibility genes that correspond with differences in prevalence [4], [17]. Additionally, thousands of unannotated transcripts have been identified within populations [18], [19], highlighting the difficulty in distinguishing population-specific transcripts that are functionally relevant versus those that simply arise from noisy splicing [20]. Elucidating how gene expression regulation and splicing are impacted by historical human migrations will aid functional interpretation of the genome and improve our understanding of the transferability and evolution of genetic regulation across populations.

This study aims to characterize regulatory, splicing, and expression differences via RNA sequencing across a global sampling of seven populations from the HGDP. We have also performed medium pass genome (∼8X) and high coverage (∼96X) exome sequencing of these individuals, enabling us to characterize genetic effects on transcriptome variation. These integrated DNA and RNA sequencing datasets are generated from the broadest points of human migration history yet sampled, and serve as a resource for future studies analyzing functional differences across populations.

## Results

**Figure 1:**
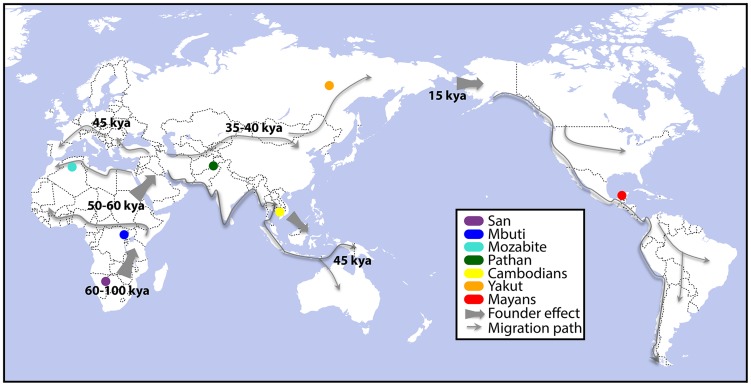
Collection sites for genome-, exome-, and RNA-sequenced human lymphoblastoid cell lines (LCLs). LCLs were immortalized from the populations highlighted above, as described previously [21], and the genomes, exomes, and transcriptomes were sequenced. Founder effect and migration paths have been reproduced from [53] to highlight the breadth of human migration history across which these LCLs were sampled.

To assess the molecular underpinnings of population level transcriptome diversity, we have sequenced the DNA and mRNA fractions of 45 lymphoblastoid cell lines (LCLs) from seven populations in the Human Genome Diversity Panel [21]: Namibian San, Mbuti Pygmies of the Democratic Republic of Congo, Algerian Mozabites, Pathan of Pakistan, Cambodians of East Asia, Yakut of Siberia, and Mayans of Mexico (Figure 1). Five of these groups are descended from the ancient human dispersals out of Africa associated with serial founder effects [5]. The populations in this study capture important differences in human genetic diversity resulting from early subdivision within Africa and subsequent serial founder effects into the Near East, back to North Africa [22], southern and eastern Asia and Central America.

DNA sequencing was performed via paired-end 101-base pair Illumina sequencing (Methods). Total coverage per individual genome and exome was 8.1±3.3X and 96.5±11.0X (mean ± standard deviation), respectively. Additionally, 15.4M±0.5M read pairs per sample were generated via transcriptome sequencing performed on lymphoblastoid polyA-selected mRNA, and an average of 10.8±4.6 million read pairs per sample were properly mapped to the hg19 transcriptome (Table 1). Gene quantification performed through Cufflinks [23] detects an average of 9,141 known genes expressed per each individual cell line, which is consistent with previous observations [12].

**Table 1:** RNA-sequencing summary.

Population	Samples	Male	Female	Mean Reads	Mean Mapped Pairs	Mean Percent Mapped	Mean Genes Expressed (>1 RPKM)
Cambodian	7	4	3	41,976,806	33,126,460	78.61%	9,630
Maya	7	2	5	37,789,223	30,512,266	79.79%	9,888
Mbuti Pygmies	7	5	2	38,707,021	29,978,422	77.05%	8,865
Mozabite	7	4	3	37,375,673	29,163,656	77.90%	9,094
Pathan	7	3	4	35,045,723	27,067,137	76.62%	8,797
San	4	4	0	37,227,991	26,472,133	71.57%	7,900
Yakut	6	3	3	34,847,733	27,182,512	78.02%	9,244
Total	45	25	30	37,650,211	29,287,092	77.43%	9,141

### mRNA quantification, reproducibility, and normalization

We randomized library preparations and sequencing across populations, including approximately one individual per population in each lane of sequencing in order to ensure that expression differences were due to biological rather than technical variation. We also sequenced technical replicates for each sample by sequencing each library preparation twice per individual. We assessed the correlation between replicates and identified problematic samples as previously described [24]. Briefly, we applied an optimal power space (OPS) transformation to expressed gene and transcript quantifications to ensure that all data points contributed equally to correlation measures, eliminating bias by low and high FPKM values. Pearson correlations between technical replicates were high *(* ***r*** = 0.915±0.034 (mean ± sd) for genes (Figure S1), ***r*** = 0.641±0.167 for transcripts). Higher correlations between replicates for gene versus transcript quantifications likely reflect the greater uncertainty in the deconvolution of the relative abundance of transcripts within a gene. Because reproducibility between replicates was high, we pooled reads across replicates and reassessed gene and transcript quantifications with Cufflinks. For each sample, we determined the median Pearson correlations (D-statistics) with all other samples. D-statistics were high overall (median D-statistic = 0.948 for genes, median D-statistic = 0.862 for transcripts, Figure S2). We identified two outliers, both within the San population (HGDP01029 and HGDP00992), and we removed these samples as well as the two remaining San samples from all downstream analyses.

To compare gene expression patterns across individuals, we first normalized our data. Exon and gene counts were quantified over regions annotated in UCSC known gene tables. Previous work has shown that the sample preparation protocol for RNA-seq introduces nonlinear, sample-specific effects that explain more than 50% of the variation in expression data [25], [26]. These nonlinear effects can manifest as sequence-specific biases [13], which we accounted for via conditional quantile normalization (CQN) [27]. This normalization strategy removed large distributional outliers (Figure S4) by accounting for non-linear guanine-cytosine (GC) content and feature length effects.

### Genetic differentiation

**Figure 2:**
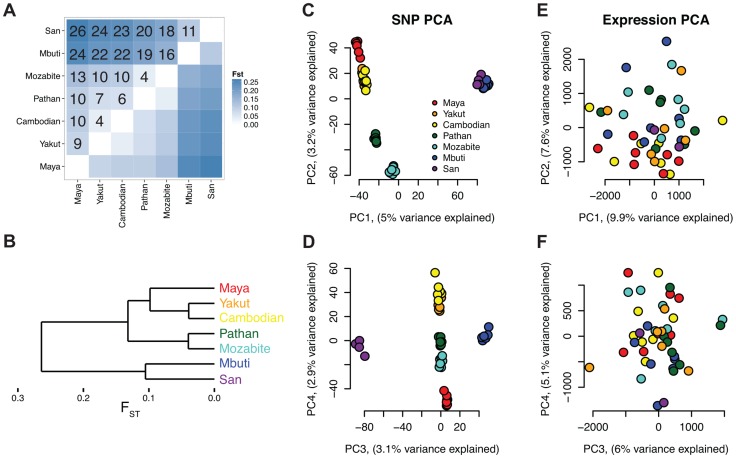
Analysis of genetic and expression divergence among individuals and populations. A) F_ST_ matrix with 100*F_ST_ values shown in the upper half and B) tree generated via hierarchical clustering. C–F) Principal components analysis (PCA) of genetic (C and D) and expression (E and F) values. Genetic values are from exome variants, which were called from high coverage (96X) sequence data.

As previously observed, genetic variation clearly differentiates globally diverse populations [28], [29] (Figure 2A–D). A tree generated via hierarchical clustering of F_ST_ distances (Figure 2A–B) shows a clear separation of sub-Saharan African populations and out-of-Africa populations. Additionally, principal component analysis (PCA) of autosomal single nucleotide polymorphisms (SNPs) in the HGDP dataset (Figure 2C–D) shows population-specific clustering [29] with these seven global populations separating within the first four PCs. Despite clear clustering among the selected populations at the genetic level, PCA of gene expression levels assessed via Cufflinks reflects high individual expression variability and shows no clear population clustering (Figure 2E–F). A formal test of this hypothesis is presented in the last subsection of the Results section, “*Variability in expression and alternative splicing ratios*,” which also considers the impact of population labeling as a factor in gene expression differences among individuals.

### Differential expression across populations

**Figure 3:**
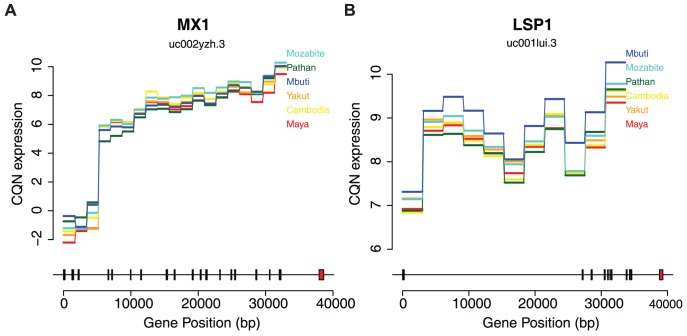
Differential expression across human populations. Bottom plots show exon positions indicated by rectangles to physical position scale. Red rectangles are differentially expressed exons. Upper plots show the median conditionally quantile normalized (CQN) expression values per population of each exon in horizontal lines. Diagonal lines connect each exon. Each exon corresponds one-to-one with the transcript structure shown below but have been scaled evenly to the width of the plot for ease of visualization. Population orders on the right correspond with the order of expression values of the last exon. A) Expression by population of the uc002yzh.3 transcript of MX1. B) Expression by population of the uc001lui.3 transcript of LSP1.

We next sought to identify individual exons and genes that show strong evidence of differential expression (DE) among populations. We used a negative binomial model for gene expression analyses (Methods) and incorporated a normalization offset term from CQN via edgeR [30] (Figure S3); we find that our model provides a good fit to the data (Methods, Figure S4). We identified 251 DE exons via generalized linear model with a false discovery rate (FDR) of less than 5% when comparing all populations (Table S1). Two examples of genes containing highly DE exons are shown in Figure 3 (expression of all individuals shown in Figure S5), both of which are involved in immune function and have some previous evidence for population-specific effects [31], [32]. Figure 3A shows the expression of MX1 colored by population (FDR = 1.57%). MX1 is known to affect the immune response to influenza, the West Nile Virus, the avian flu, and other DNA and RNA viruses [33], [34]. Additionally, LSP1 (lymphocyte-specific protein 1, Figure 3B, FDR = 0.87%) has been associated with breast cancer risk in Europeans. Interestingly, this signal did not replicate using admixture mapping in Latina women, perhaps due to differences in allele frequency among the GWAS and attempted replication populations [32]. We also identified 44 differentially expressed transcripts at ≤5% FDR (Table S2). We used gene set enrichment analysis (GSEA) of ranked p-values to detect functional enrichment of differentially expressed transcripts [35]. The following categories were enriched with a FDR≤5%: RXR and RAR heterodimerization with other nuclear receptors (q = 0.007, canonical pathway), IL 2 signaling pathway (q = 0.015, BioCarta), and Top 40 genes from cluster 7 of acute myeloid leukemia (AML) expression profile (q = 0.018, chemical and genetic perturbations) (Figure S6).

### Allelic variation in expression

Allele-specific expression (ASE) can be detected as a read imbalance at a given heterozygous site; it has previously been shown to tag regulatory variants [12]. To identify the degree to which allelic effects on expression vary, we compared ASE sharing among individuals for variants in the high coverage exomes. We define normalized ASE sharing as the number of shared significant ASE events (p<0.05) with at least 30 reads, normalized by sharing of SNPs that are heterozygous with at least 30 reads, regardless of presence or absence of a significant allelic imbalance. Reads were sampled to have equal counts in order to account for expression variability. There is a rapid reduction in normalized ASE sharing as the number of individuals in the comparison set increases (Figure S7A). That is, even when heterozygous sites are shared, most allelic imbalances are private to an individual. Some allelic imbalances are shared by pairs of individuals; rarely do three individuals in the set share an imbalance and very little sharing occurs across more than four individuals. We compared normalized ASE sharing across individual pairs and found similar levels of sharing within and between populations (Figure S7B). A potential explanation for this lack of ASE sharing among individuals is that the allelic state of the underlying causal regulatory variant tagged by the ASE exome site is acting in *cis* but in weak linkage disequilibrium, potentially with a rare regulatory variant.

In a previous study, Stranger *et al.* (2012) mapped eQTLs in eight populations from the HapMap3 dataset. To determine if the effects of these previously identified *cis*-regulatory variants can be captured in our more diverse HGDP populations, we compared ASE events in our dataset to previously discovered eQTLs [3] across populations. We hypothesized that if an individual is heterozygous for a previously discovered *cis* eQTL SNP (eSNP), and a significant ASE signal exists in the associated gene, then the allelic imbalance is more likely to be driven by the eQTL (see Figure S8 for a graphical representation of the model). We assessed the HGDP genotypes of eSNPs identified in HapMap3 and determined that there is a significant ASE enrichment within eQTLs associated with heterozygous versus homozygous eSNPs (p<2.2×10^−16^, Figure S9). This finding is consistent with our model and previous studies [12] and indicates that our measures of ASE are tagging shared regulatory variation between these studies. We also calculated an enrichment score similar to an odds ratio to determine how often ASE events are found in heterozygous versus homozygous eQTLs compared to the number of measured sites (Methods) for each HGDP and HapMap3 population. We observe an enrichment of ASE events in heterozygous eQTLs versus homozygous eQTLs consistently in all populations, but we do not observe a signal showing stronger effects in HGDP populations that are more closely related to the eQTL discovery population (Figure S10). This supports the previous notion that the effects of common regulatory variation are largely shared across populations with taggability depending on patterns of shared LD [3].

**Figure 4:**
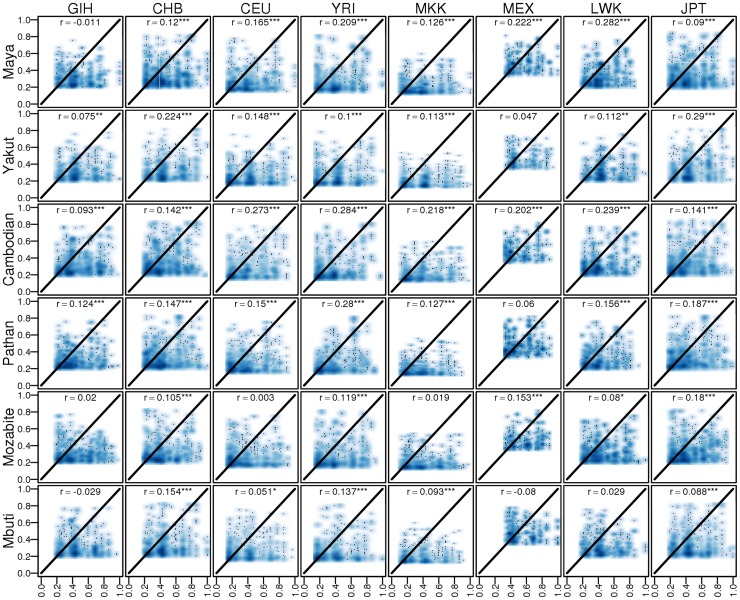
Comparison between eQTL correlations (ρ^2^) discovered in HapMap3 vs replicated in HGDP. * indicates p<0.05, ** indicates p<0.01, *** indicates p<0.001. ρ^2^ values in this dataset were filtered to the same minimum threshold as in the HapMap3 study for consistency.

We next sought to determine whether regulatory events discovered within populations replicate more consistently in more closely related populations. Because of the limited sample size and structured populations in this study, *de novo* eQTL discovery is infeasible. We therefore assessed cross-population regulatory sharing using previously discovered eQTLs [3]. We compared Spearman's rank correlation coefficient ρ^2^ values, a measure akin to variance explained, between our dataset and the HapMap3 study and find consistency between the associations (*r* = 0.22, p<2.2 * 10^−16^). The −log_10_(p) values across studies were also significantly correlated (*r* = 0.14, p<2.2 * 10^−16^). We next measured the associations between eQTLs identified in each population. We find that the effect sizes of eQTLs are significantly associated across most pairwise populations (Figure 4), independent of genetic divergence. The reproducibility of eQTLs is similar across populations, indicating that previously discovered common eQTLs reflect either the true causal SNPs or tag the causal eQTL due to similar LD at the locus (Figure S11). We also assessed the impact of similarity in allele frequencies between studies on the ρ^2^ values and find that eQTLs with similar minor allele frequencies (MAFs) between studies replicate better than eQTLs with different minor allele frequencies. As expected, eQTLs with high MAFs in one study and low MAFs in another study replicate poorly (Figure S12).

### Novel transcribed regions across populations

Using the genome, exome, and RNA-seq resource described above, we characterize the completeness of current gene annotations as previously described [13]. By pooling our dataset of 1.7 billion paired reads, we identify regions of novel transcription that lie outside of previously characterized gene structures. By calculating per-base global sequencing coverage and merging together continuous transcribed regions above our cutoff filters, we identified 445,091 total regions of putative transcription in our LCLs, 384,285 (∼86%) of which corresponded to annotated exons in Refseq, Ensembl, UCSC, or Gencode databases (Methods, Figure S13). Conversely, 34,555 regions (∼7%) meeting our minimum expression threshold did not overlap with known annotations (Figure S14).

When we filter regions expressed in at least one individual per population at greater than or equal to 1 RPKM, there are only a few hundred of these 34,555 regions expressed across all individuals in that population (Table S3). Additionally, we see that every novel transcribed region expressed ubiquitously in one population is also present in at least one other individual of another population. This result suggests that the vast majority of novel transcribed regions are not population specific, but can be found across multiple diverse human groups.

### Variability in expression and alternative splicing

Previous work indicates that exonic splicing may vary significantly more than gene expression variability across species within the same tissue [36], [37]. The majority of previous human transcriptome work has focused on expression and regulatory variability, leaving the degree of alternative splicing variation across diverse human populations relatively unexplored. To understand expression and splicing relationships within and between human populations, we measured the coefficient of variation, *cv*, in gene expression (standard deviation divided by the mean) and the variability in alternative splicing ratios (Hellinger distance to the centroid of the splicing ratios of each gene across all individuals in the population, 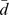) using methods developed previously [4]. We find that the *cv* and 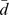 values for genes are highly correlated between pairwise populations (*cv* correlations are, on average, within [0.44, 0.67] between pairwise populations, p<2.2×10^−16^ for each comparison (Figure S15), and 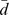 correlations are on average within [0.64, 0.82] between pairwise populations. p<2.2×10^−16^ for each comparison (Figure S16)). The relationships overall between *cv* and 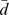 values do not reflect the genetic divergences seen between pairwise populations (Mantel test with 1,000 Monte Carlo repetitions between *cv* Spearman rank correlation distance matrix and F_ST_ gives ρ = 0.38, p = 0.16, and the same test between 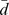 and F_ST_ gives ρ = 0.44, p = 0.14).

**Figure 5:**
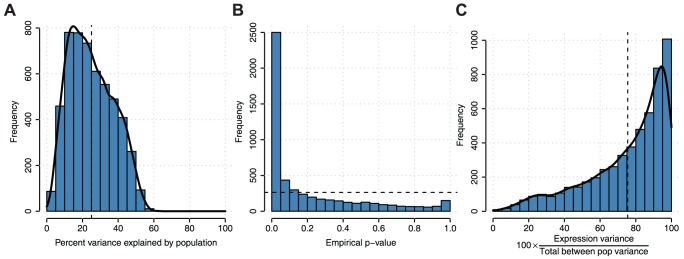
Analysis of variation in gene expression and splicing among individuals attributable to population labels. A) Distribution of percent variance explained by population across n = 5,334 expressed genes with ≥2 transcripts expressed across all individuals. B) Empirical p-values for genes in part A. P-values were calculated by permuting population labels for individuals 100 times and comparing to true population labels. Dashed line indicates the uniform p-value distribution expected under the null hypothesis of no association between population label and expression. The output from the multiplicative model can be interpreted similarly to an R^2^ coefficient of a linear model. C) The contribution of gene expression in the variance explained by the population.

**Figure 6:**
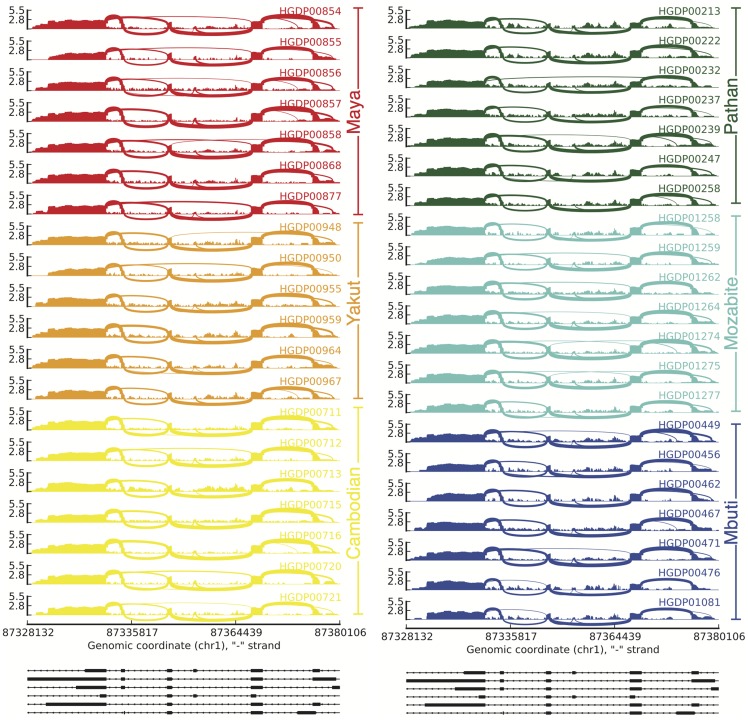
Splicing variability across human populations. Each sashimi plot shows the expression within an individual for a differentially spliced gene (ENSG00000183291.11, SEP15). The RNA-seq read densities supporting expression over the region as well as the inclusion and exclusion of exons are shown and line densities are proportional to reads supporting splicing events. The y-axis on each sashimi plot indicates the expression in log_10_ reads per kilobase per million reads (log_10_(RPKM)). The plots on the bottom show the transcript structure within the gene.

We next used established methods to assess the proportion of gene expression variation among individuals attributable to population identity [1]. We find that population label, on average, explains 25.0% of the variation in gene expression among individuals (Figure 5A) for all genes expressing at least two transcripts. To assess significance for each gene, we used a permutation test reshuffling population labels among individuals and find that the p-value distribution is heavily skewed towards low p-values compared to the expected uniform distribution (Figure 5B). This genome-scale level of population stratification for gene expression is higher than previously seen by the GEUVADIS consortium [1], which reported ∼3% of the variance attributable to population label as a factor when considering populations of mostly European descent in the 1,000 Genomes Project. These results are perhaps expected given that the populations in our study span a greater breadth of human genetic diversity. We repeated this analysis comparing each population to all other populations and find that a smaller proportion of the variation is due to population-specific differences and that these differences do not follow the pattern expected by population divergence (Figure S17). We also decomposed population-specific variability into variability in overall expression levels as opposed to splicing variability via multiplicative model, which, as previously demonstrated [1], [4], accounts for differences in scales and units between expression and splicing metrics. We find that on average, variation in gene expression explains the majority (75.5%±22.3% (mean ± sd), Figure 5C) of population-specific variation, indicating that alternative splicing generally makes up the minority of population-specific variation within humans. We repeated this analysis comparing each population to all other populations and find consistent results (Figure S18). We next assessed differential splicing between pairwise populations. In Figure 6, we show a sashimi plot of a gene (ENSG00000183291.11, SEP15) with substantial differential splicing across all pairwise populations. Overall, we do not see evidence for differential splicing patterns consistent with population genetic divergence (Figure S19); this result is consistent with a minority of population-specific variance in gene expression levels explained by splicing variability.

## Discussion

We have analyzed the transcriptome landscape from populations spanning the breadth of human genomic diversity. While other studies have characterized variation within and among populations [12], [13], this study provides a unique opportunity to discover regulatory drivers of expression diversity in serially bottlenecked populations throughout human migration history. The HGDP populations in this study were explicitly chosen to encompass a large geographic range that experienced varied demographic histories, and thus they provide unique insight into global variation in transcription. In addition to gaining an understanding of transcriptome variation in diverse populations, this study also enables the discovery of novel gene structures and provides a public resource for analyses of diverse human transcriptomes.

In this study, we have assessed population-specific expression variability, alternative splicing, and regulatory variation. We account for technical artifacts in our analyses, including GC content and feature length effects, which otherwise add nonlinear systematic noise to expression data. We show that we substantially reduce technical sources of variation from these effects in our data and obtain high reproducibility between sequencing replicates. We detect few differentially expressed exons, which is likely affected by the fact that we analyze cultured cell lines grown in a highly homogenous environment. Further, given our sample size per population, we are only powered to detect very dramatic differences in expression among populations. Using variance decomposition methods developed previously, we find that 25.0% of transcription variability can be attributed to population differences among the six we study here. A previous study that sought to detect expression differences between the CEU and YRI estimated that ∼17% of genes were differentially expressed across these populations [38]. This estimate is quite comparable to ours. However, the estimates from both studies are substantially larger than those reported by the GEUVADIS consortium, which found that population labels accounted for only ∼3% of transcription differences among 462 individuals sampled from the European populations in the 1000 Genomes Project as well as Yorubans. One potential reason why our analysis produced estimates larger than GEUVADIS is that the European populations sampled there are more closely related to each other than the breadth of populations studied here.

Immunity genes as a whole are overrepresented in the set of differentially expressed genes across populations. This is highly consistent with the immune role of LCLs we study here. This finding is also consistent with previous work showing that natural selection may have favored different alleles in certain immune genes across human populations and that differences in autoimmune disease risks may be a side consequence of differences in these evolutionary histories [39], [40]. The increased expression of immune genes in LCLs also improves our power to detect differences with respect to most other gene functions. Potential mechanisms for differential expression across populations include variation in *cis* and *trans* eQTL allele frequencies, environmental differences, and epigenetic differences.

We also measured the population-specific variance attributable to expression versus splicing and find that on average, 75.5% is due to gene expression differences. This result is consistent with previous findings in humans and indicates that, within tissues, splicing differentiates populations less than expression. While this finding is consistent with previous human studies [1], [4], it appears to be inconsistent with other cross-species work [36], [37]. This suggests that splicing potentially plays a greater role on longer evolutionary time-scales. Additionally, the methodology used to assess splicing varies substantially between these studies; in this study, we have used variance decomposition methods relying on gene and transcript annotation data, which is more limited in many other species. In the cross-species studies, exonic splicing was measured via “percent spliced in” (Ψ), which may be affected by expression variability or other forms of transcript differences, such as those arising from alternative start sites. Further work on the efficacy of alternative splicing quantification methodologies would benefit future studies.

We also show that eQTLs that were previously identified across a wide range of human populations show allelic imbalances and replicate consistently across populations, but this replication is dependent on minor allele frequencies. Our results suggest that rare eQTLs within a population that are common in another population will likely have differing effect sizes. Given that the ∼1.2 million SNPs assayed in HapMap3 are common and therefore largely shared globally, we have only limited power to assess the effects of rare regulatory variants. As more transcriptomes are sequenced across diverse populations, we expect that rarer eQTLs identified in large population-based genome- and RNA-sequencing studies will identify more population-specific enrichment patterns.

This study provides the first analyses of transcriptome diversity from serially bottlenecked populations spanning the breadth of human migration history. In this study, we integrated genome, exome, and transcriptome sequencing data from LCLs that are part of the HGDP. This enabled us to assess regulatory drivers of global expression variation in serially bottlenecked populations across a large geographic range and different demographic histories. We find that population of origin accounts for ∼25% of variation in transcription. While we are powered to detect only large differences in expression among populations, genes involved in immunity are overrepresented in this set. Of the 25% difference in transcription explained by population of origin, expression differences accounts for three-fold more of the effect than do splicing differences. Further, the common regulatory variants we replicate here impact expression across broad geographic groups relatively uniformly and do not correlate with the degree of genetic divergence among populations. We look forward to larger studies spanning the breadth of human diversity that are better powered to detect additional population-specific effects and cellular mechanisms of global expression variation. Here, we analyze the total variance in expression and splicing explained by global populations, which, together with other studies, suggests a complex genetic mechanism for population level variation in transcription.

## Materials and Methods

### RNA preparation, library construction and sequencing

Total RNA was extracted from lymphoblastoid cell lines in four San, seven Mbuti Pygmies, seven Mozabites, six Pathan, seven Cambodians, seven Yakut, and seven Mayans from the Human Genome Diversity Panel using an RNeasy Mini Kit (Qiagen). mRNAs were purified using magnetic oligo-dT beads and randomly fragmented to 300–400 nucleotides in length. First-strand cDNA synthesis was performed using random hexamers and reverse transcriptase. This was followed by second-strand cDNA synthesis with dUTP via the dUTP strand-marking protocol [41]. Illumina TruSeq adaptors were ligated to the ends of the double-stranded cDNA fragments followed by digestion with uracil N-glycosylase (UNG) to remove second strand cDNA. A 300–400 bp size-selection of the final product was performed by gel-excision, following the Illumina-recommended protocol.

Each individual was sequenced in a 7-plex library on an Illumina HiSeq 2000 producing 101-bp paired end reads. Lanes were assessed for multiple quality metrics including number of reads, read quality, and reads mapping to the human genome. Two San individuals failed sequencing quality control and so all four San individuals were excluded from further analysis.

### Exome capture

Sample genomic DNA was extracted from lymphoblastoid cell lines. Exonic regions were enriched using an Agilent SureSelect ^XT^ 44 Mb All-Exon Capture Kit (v2) and sequenced on Illumina HiSeq machines.

### Exome and genome read mapping and SNP calling

Illumina sequencing reads were mapped to the human reference genome (hg19) using a standard pipeline informed by the 1000 Genomes Project [42]. Briefly, reads were mapped and paired using bwa v0.5.9 [43]. Duplicate read pairs were identified using Picard (http://picard.sourceforge.net/). Base qualities were empirically recalibrated, indels were realigned, and variants were called using the Genome Analysis Tool Kit (GATK) v1.6 [44]. SNP calls that failed the Variant Quality Score Recalibration (VQSR) step were filtered out.

### F_ST_ calculations

Exonic SNPs were annotated using the RefSeq database to identify synonymous coding variants. High confidence and high coverage synonymous variants were used to compute Weir & Cockerham F_ST_ values [45] for each pairwise population using vcftools (v0.1.11) [46].

### RNA sequencing read mapping

Reads were mapped to the human reference genome (hg19) with bowtie-2.0.0 and tophat-2.0.4 split read mapping algorithms using the “-b2-very-sensitive” parameters [46]. Reads were subsequently filtered to include only properly paired reads. This yielded between 12.1 and 44.8 million reads per individual (29.3 mean±7.9 s.d. million reads), which corresponds to 62.17±13.79% of the total reads per individual.

### Quantification and normalization of known exons and genes

Exon and gene count estimates were created by using bedtools to count read overlap with known genes and exons from the UCSC “knownGene” table file downloaded on July 17th, 2012 for differential expression analysis. Raw exon and gene read counts were normalized through conditional quantile normalization, which reduces expression outliers by accounting for feature level GC nucleotide content and overall feature length [27].

UCSC knownGene tables were also used for novel transcript structure analysis because a larger collection of gene structures have been catalogued in this annotation set. For all other analyses, gencode v13 annotations were used, because they give one-to-one correspondence of transcript to gene annotation, enabling the Gonzalez-Porta methods to be used as they were developed.

### Quantification of known transcripts

Transcript level quantification was performed with cufflinks-2.0.2 and produced FPKM (fragments per kilobase of exon per million) estimates per transcript. Cufflinks uses a generative statistical model of paired-end sequencing experiments to derive a likelihood for the abundances of a set of transcripts given a set of fragments. The likelihood function can be shown to have a unique maximum, which Cufflinks finds using a numerical optimization algorithm. The program then multiplies these probabilities to compute the overall likelihood that one would observe the fragments in the experiment, given the proposed abundances on the transcripts [23].

In order to compare expression levels in this dataset with those identified in Stranger et al [3], we reran Cufflinks (v2.1.1) using the Gencode v13 annotations to get both gene and transcript quantifications. These expression abundances were subsequently used to quantify the relative importance of variability in gene expression and variability in alternative splicing to individual transcript variability.

### Annotation of genetic variants

Sequencing variants called from the differentially expressed and differentially spliced regions were annotated for a series of functional predictions, conversation scores, and RefSeq database annotations as described below. This was done in order to better assess the significance of genetic variants present in the data and their potential contribution or involvement in modulating gene expression, transcript splicing, and phenotypic variability. General annotations include information from: the NHLBI Exome Sequence Project allele frequencies; 1000 Genomes Project allele frequencies; publically available Complete Genomics sample allele frequencies; region and exonic annotations from both Ensembl and RefGene; and information about protein structure and function from the UNIPROT and INTERPRO databases. Conservation scores were also produced from the following algorithms: GERP++, SLR, SIFT, LRT, PHYLOP, and SiPhy based on 29 mammalian genomes [47]–[51]. Lastly, functional prediction annotations were produced from the following sources: FATHMM, MutationTaster, Mutation Assessor, LRT, PolyPhen2, and the RefSeq RefGene database [50], [52].

### Identifying unannotated transcription

Methods to characterize regions of previous unannotated transcription closely followed previously described work [13] (Figure S14). In brief, for each base of the genome we calculated global sequencing coverage and split the genome into continuous transcribed regions. Expression of a region was defined as the maximum per base coverage of bases in the region. As in previous studies, we chose a threshold of an average expression level of 5×10∧-8 (or 0.05 reads/million) to consider a region expressed and merged together regions separated by less than 15 bp [13]. Sample specific expression of these novel regions was then quantified by calculating RPKM of each region for each individual. For these analyses, we ran Cufflinks (v2.0.2) using the UCSC KnownGene tables downloaded on July 16, 2012 because there were fuller annotations than in Gencode v13.

### Allele-specific expression (ASE)

ASE was determined as previously [12]. Briefly, variants were called for all HGDP individuals in this project using high coverage, high quality exome variant calls generated according to the GATK best practices. Samtools was used to determine the number of reads that matched the reference and non-reference allele. Imbalance reference allele mapping bias was compensated using the per individual overall reference ratio within the binomial test.

### Differential expression

We used conditional quantile normalization for all exons and genes with unique start and stop positions, accounting for GC content and length as covariates, and generated an offset term per gene or exon and individual. We filtered to exons or genes where the standard FPKM expression was > = 2 and the length was at least 100 bp, which left 207,180 of all UCSC knownGene annotated exons (29.7%) and 72,931 of all annotated genes (26.8%). Then, we used the following negative binomial model to detect differential expression:
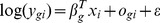
Here, *y* is the count at gene *g* in individual *i*, *β* is the vector of population effects, *x* is the population label, *o* is the offset term from conditional quantile normalization, and ε is the error term. We perform an analysis of variance (ANOVA) comparing the null hypothesis of *β* = 0 to the alternative hypothesis of *β*≠0. In pairwise population comparisons, we computed genewise exact tests for differences in the means between the two groups of negative-binomially distributed counts.

### ASE enrichment within eQTLs



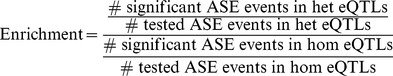
eQTLs discovered in the HapMap3 populations were replicated in our HGDP dataset using genotypes derived from the exome sequencing variants and preliminary results for the full genomic variants (Henn & Botigue et al, unpublished data) for eQTLs outside the exome (Data Access).

### Data access

The SRA accession number for the genome and exome sequence data reported in this paper is SRP036155. The GEO accession number containing the RNA-Seq data and gene/transcript expression matrices reported in this paper is GSE54308. Links to additional data (exome variant files, eQTL SNP data, F_ST_ matrices, gene/transcript expression quantifications, ASE tables, and eQTL data) and scripts are provided on an FTP site by the Stanford Center for Genomics and Personalized Medicine computing cluster located here: http://bustamantelab.stanford.edu/datasets.html.

## Supporting Information

Figure S1 Reproducibility across all samples between two sequencing replicates for each sample. An optimal power space (OPS) transformation has been applied to the gencode FPKM values for expressed genes for each sample. A linear regression line is shown as a red dashed line in each plot. The x-axis corresponds to the OPS-transformed FPKM values corresponding with the first run and the y-axis corresponds to the OPS-transformed FPKM values corresponding with a second run.
(TIF)

Figure S2 Test for outliers. Histogram of median pairwise Pearson correlations (D-statistics) between gene and transcript expression levels after OPS transformation.
(EPS)

Figure S3 Expression distribution pre- and post- normalization. Each line represents the expression distribution for a single individual. A) Standard fragments per kilobase per million reads (FPKM) expression distribution. B) Conditional quantile normalization (CQN) expression distribution accounting for guanine-cytosine (GC) content and exon length effect QR fits shown in C–D, as described in Hansen, Irizarry, & Wu, 2012. C) QR fit of GC content to read counts via B-spline. D) QR fit of exon length to read counts via B-spline. C–D) Knots on the x-axis indicate the 2.5%, 25%, 50%, 75%, and 97.5% quantiles of the data.
(EPS)

Figure S4 Q-Qplot of goodness of fit statistics using empirical Bayes dispersions calculated in edgeR. Fit statistics were calculated as described in Figure 2 of [30].
(TIF)

Figure S5 MX1 and LSP1 CQN expression levels, which contain differentially expressed exons. These correspond with Figure 3, but also show the expression levels for each individual in addition to the thicker lines (which show the medians). As before, bottom plots show to scale exon positions indicated by rectangles. Red rectangles are differentially expressed exons. Upper plots show the median conditionally quantile normalized (CQN) expression values per population of each exon in horizontal lines. Diagonal lines connect each exon. Each exon corresponds one-to-one with the transcript structure shown below, but has been scaled evenly to the width of the plot for ease of visualization. Population orders on the right correspond with the order of expression values of the last exon.
(EPS)

Figure S6 Gene set enrichment analysis (GSEA) plots for significantly enriched categories. Top portions of plots show the running enrichment scores (ES) for gene sets as the analysis walks down the ranked list. The middle portions of the plot shows where the members of the gene set appear in the ranked list of genes. The bottom portion of the plot shows the values of the ranking metric as you move down the list of ranked genes. More details can be found in the GSEA user guide. A) Enrichment plot for curated GSEA category PID_RXR_VDR_PATHWAY, defined by RXR and RAR heterodimerization with other nuclear receptors. B) Enrichment plot for curated GSEA category PID_BIOCARTA_IL2_PATHWAY, defining genes involved in the IL2 signaling pathway. C) Enrichment plot for curated GSEA category VALK_AML_CLUSTER_7, defined by the Top 40 genes from cluster 7 of acute myeloid leukemia (AML) expression profile; 61% of the samples are FAB M1 or M2 subtype.
(TIF)

Figure S7 ASE sharing. In all cases where sites had at least 30 reads, reads were downsampled to 30 and the difference from the overall reference ratio for each individual (close to 0.5 for all samples) was assessed via binomial p-value so quantify significant imbalances. A) Percentage of significant ASE sites covered by at least 30 reads shared between pairs of individuals within a population, normalized by sharing of heterozygous sites covered by at least 30 reads, regardless of imbalance. B) Clustering of normalized ASE sharing between pairs of individuals. Colors represent the population each individual belongs to.
(EPS)

Figure S8 Model for the influence of cis-eQTLs on ASE sites with homozygous reference, heterozygous, and homozygous non-reference eQTLs.
(EPS)

Figure S9 Ratio of significant ASE to measured ASE sites depending on eSNP genotype. Red line shows the ratio of significant to measured allelic imbalances in heterozygous eQTLs. Blue line shows the same scenario within homozygous eQTLs.
(EPS)

Figure S10 Comparison of eQTLs in HapMap3 vs ASE enrichment in HGDP. Enrichment in ASE is defined as the ratio between the number of significant/tested ASE events in heterozygous eQTLs divided by the number of significant/tested ASE events in homozygous eQTLs. ASE events are considered significant if p<0.05. The dashed line is drawn at 1, indicating no enrichment of ASE events in heterozygous eQTLs with respect to ASE events in homozygous eQTLs.
(EPS)

Figure S11 Linkage disequilibrium (LD) decay across population. Full exome sequencing data (196,663 SNPs) were used to assess linkage within each population.
(EPS)

Figure S12 Reproducibility of eQTLs stratified by minor allele frequency (MAF). As in Figure 4, the x- and y-axes for each subplot are the ρ^2^ values for each study. The x- and y-axes of the grid correspond to the binned study MAFs.
(TIF)

Figure S13 Novel transcribed regions analysis workflow.
(EPS)

Figure S14 Coverage distributions of novel transcribed regions. Stratifications are by A) population and B) individual.
(EPS)

Figure S15 Variability in gene expression across pairwise populations, as measured by the coefficient of variation (***cv***) which is a measure of gene expression dispersion. Only genes that passed our filters (genes expressed in all individuals, N = 5,334) were included here.
(TIF)

Figure S16Variability in alternative splicing across pairwise populations, as measured by 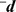, i.e. the mean Hellinger distance to the centroid of the relative abundances of alternative splice forms [4].(TIF)

Figure S17 Percent variance in gene abundance explained by each population compared to other populations in this study. Dashed black lines indicate average value.
(EPS)

Figure S18 Percentage of population-specific variance in gene expression due to overall expression levels as opposed to alternative splicing.
(EPS)

Figure S19 Tests for differential splicing across pairwise HGDP populations using the Anderson method developed in Gonzalez-Porta et al. Grey dashed lines indicate the expected null p-value distribution under the null hypothesis.
(EPS)

Table S1 Differentially spliced exons. Significant at the FDR<5% threshold. FC is fold change, CPM is counts per million, and LR is likelihood ratio.
(XLSX)

Table S2 Differentially spliced transcripts. Significant at the FDR<5% threshold. FC is fold change, CPM is counts per million, and LR is likelihood ratio.
(XLSX)

Table S3 Novel transcribed region metrics across populations.
(XLSX)
