# Supplementary figures and images for: Transcriptome Sequencing from Diverse Human Populations Reveals Differentiated Regulatory Architecture
Source: PLoS Genet. 2014 Aug 14;10(8):e1004549. doi: 10.1371/journal.pgen.1004549 (PMC4133153; doi:10.1371/journal.pgen.1004549)

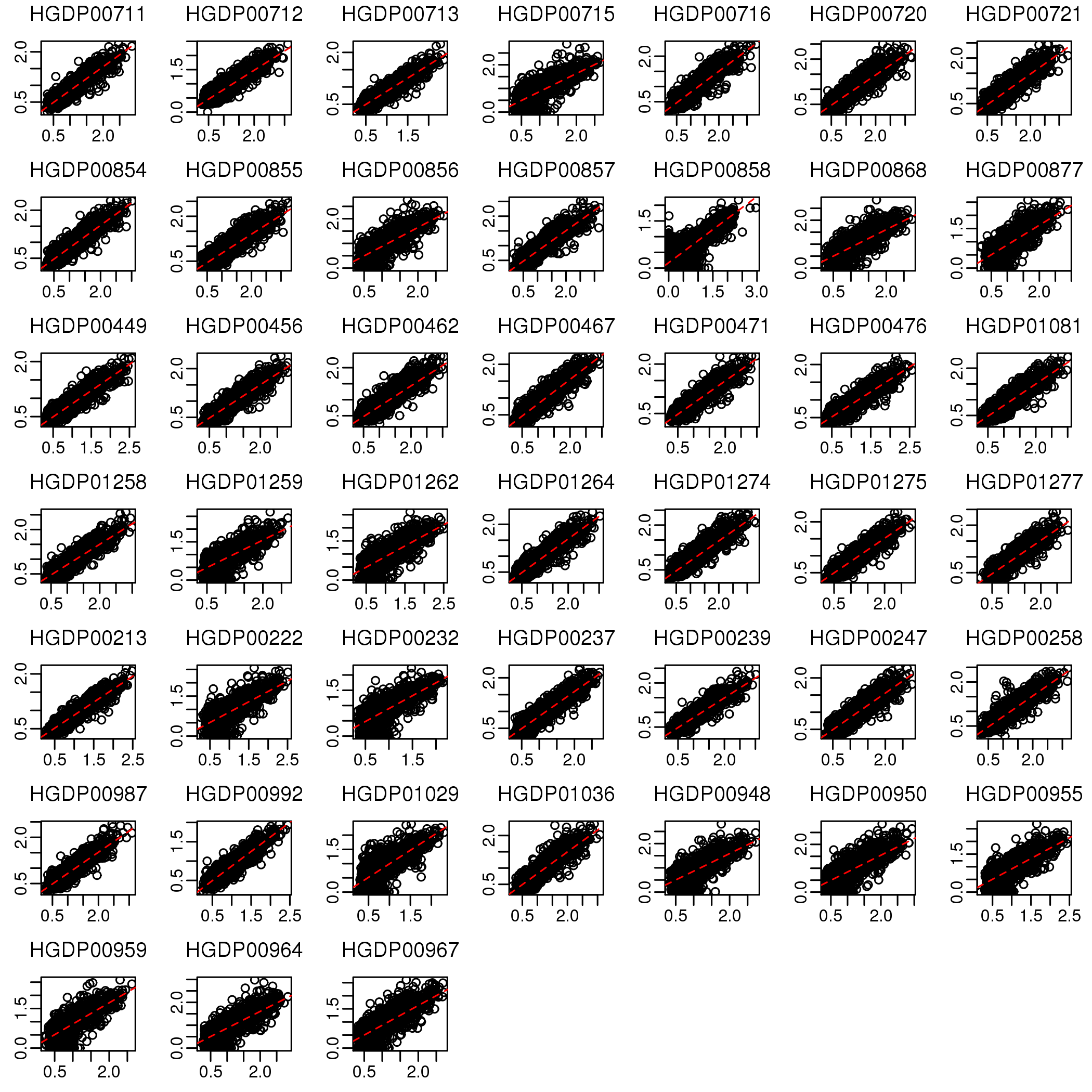

Supplement: Figure S1 — Reproducibility across all samples between two sequencing replicates for each sample. An optimal power space (OPS) transformation has been applied to the gencode FPKM values for expressed genes for each sample. A linear regression line is shown as a red dashed line in each plot. The x-axis corresponds to the OPS-transformed FPKM values corresponding with the first run and the y-axis corresponds to the OPS-transformed FPKM values corresponding with a second run. (TIF) [file pgen.1004549.s001.tif]

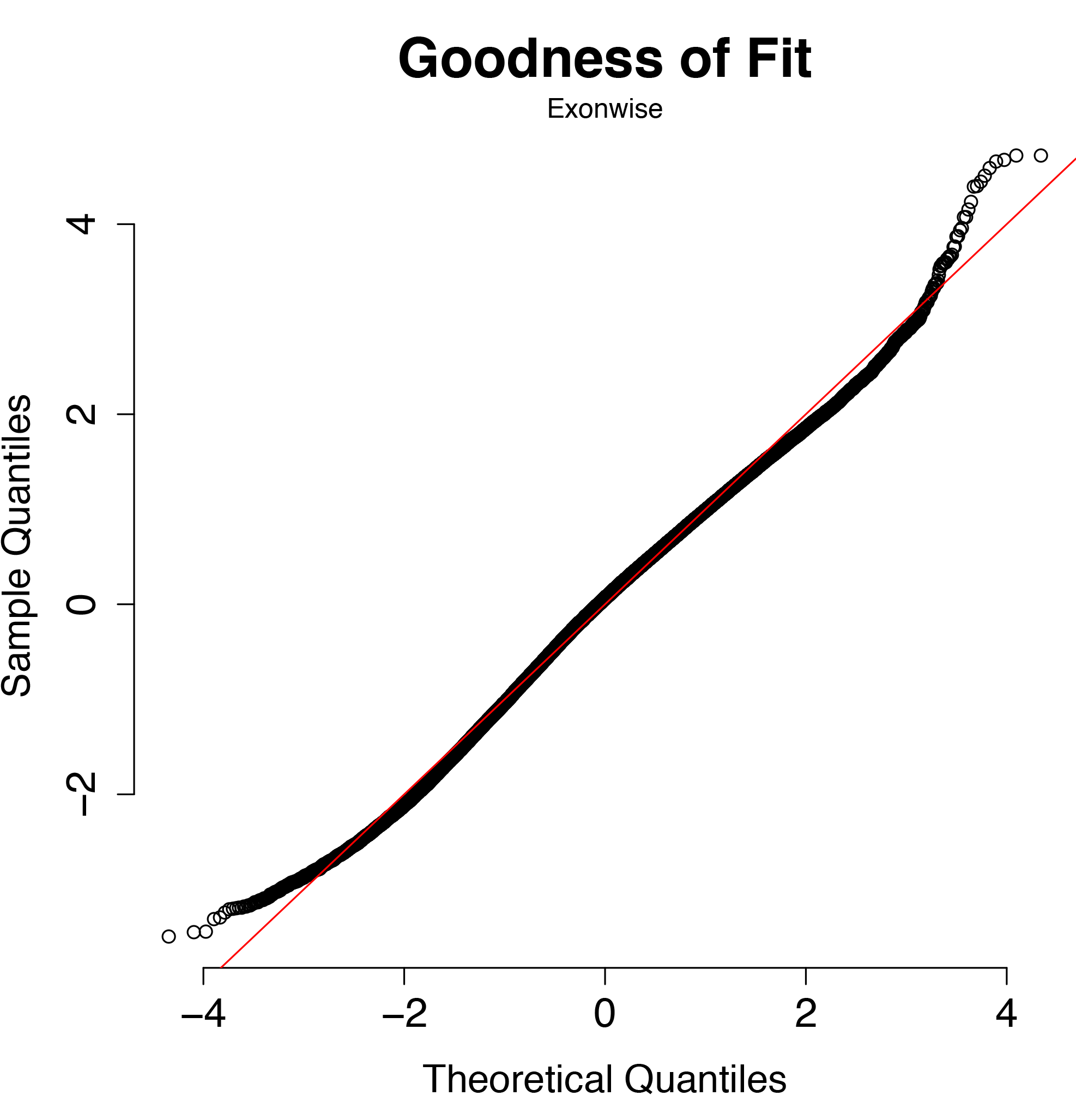

Supplement: Figure S4 — Q-Qplot of goodness of fit statistics using empirical Bayes dispersions calculated in edgeR. Fit statistics were calculated as described in Figure 2 of [30]. (TIF) [file pgen.1004549.s004.tif]

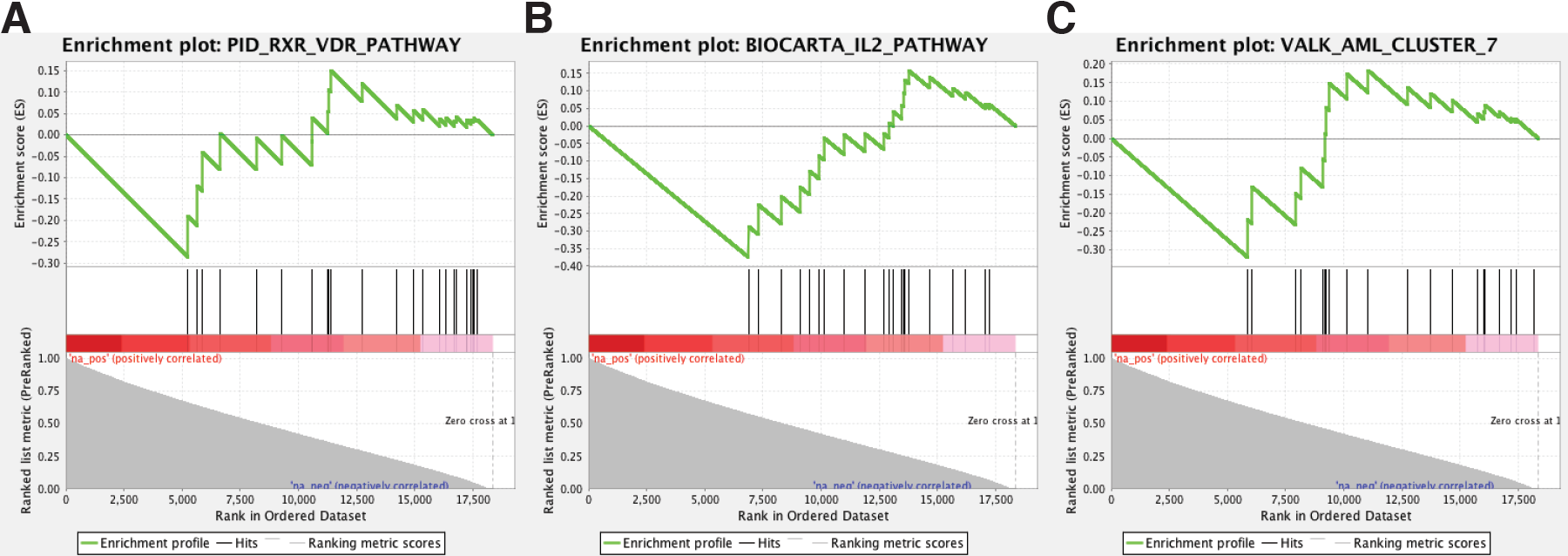

Supplement: Figure S6 — Gene set enrichment analysis (GSEA) plots for significantly enriched categories. Top portions of plots show the running enrichment scores (ES) for gene sets as the analysis walks down the ranked list. The middle portions of the plot shows where the members of the gene set appear in the ranked list of genes. The bottom portion of the plot shows the values of the ranking metric as you move down the list of ranked genes. More details can be found in the GSEA user guide. A) Enrichment plot for curated GSEA category PID_RXR_VDR_PATHWAY, defined by RXR and RAR heterodimerization with other nuclear receptors. B) Enrichment plot for curated GSEA category PID_BIOCARTA_IL2_PATHWAY, defining genes involved in the IL2 signaling pathway. C) Enrichment plot for curated GSEA category VALK_AML_CLUSTER_7, defined by the Top 40 genes from cluster 7 of acute myeloid leukemia (AML) expression profile; 61% of the samples are FAB M1 or M2 subtype. (TIF) [file pgen.1004549.s006.tif]

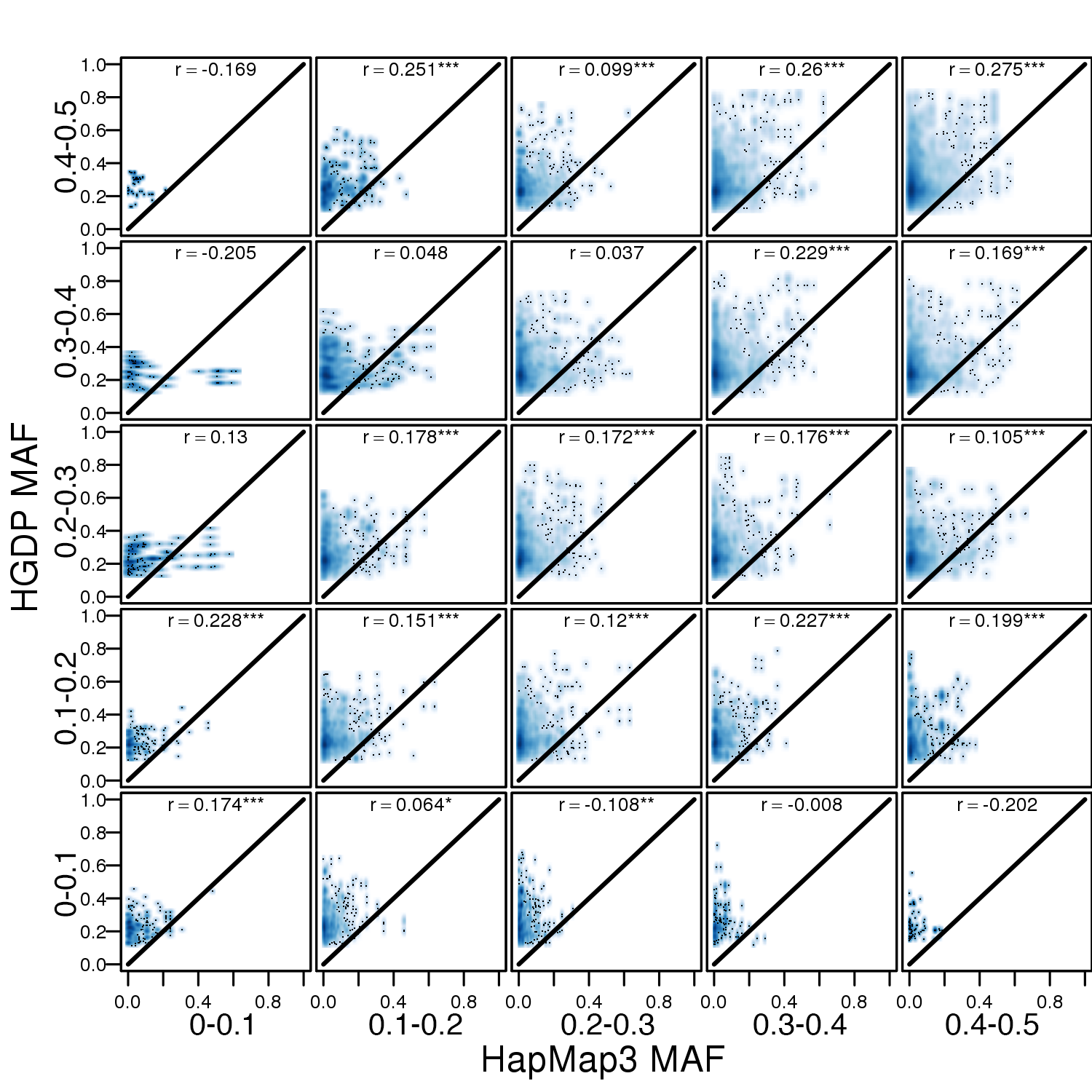

Supplement: Figure S12 — Reproducibility of eQTLs stratified by minor allele frequency (MAF). As in Figure 4, the x- and y-axes for each subplot are the ρ2 values for each study. The x- and y-axes of the grid correspond to the binned study MAFs. (TIF) [file pgen.1004549.s012.tif]

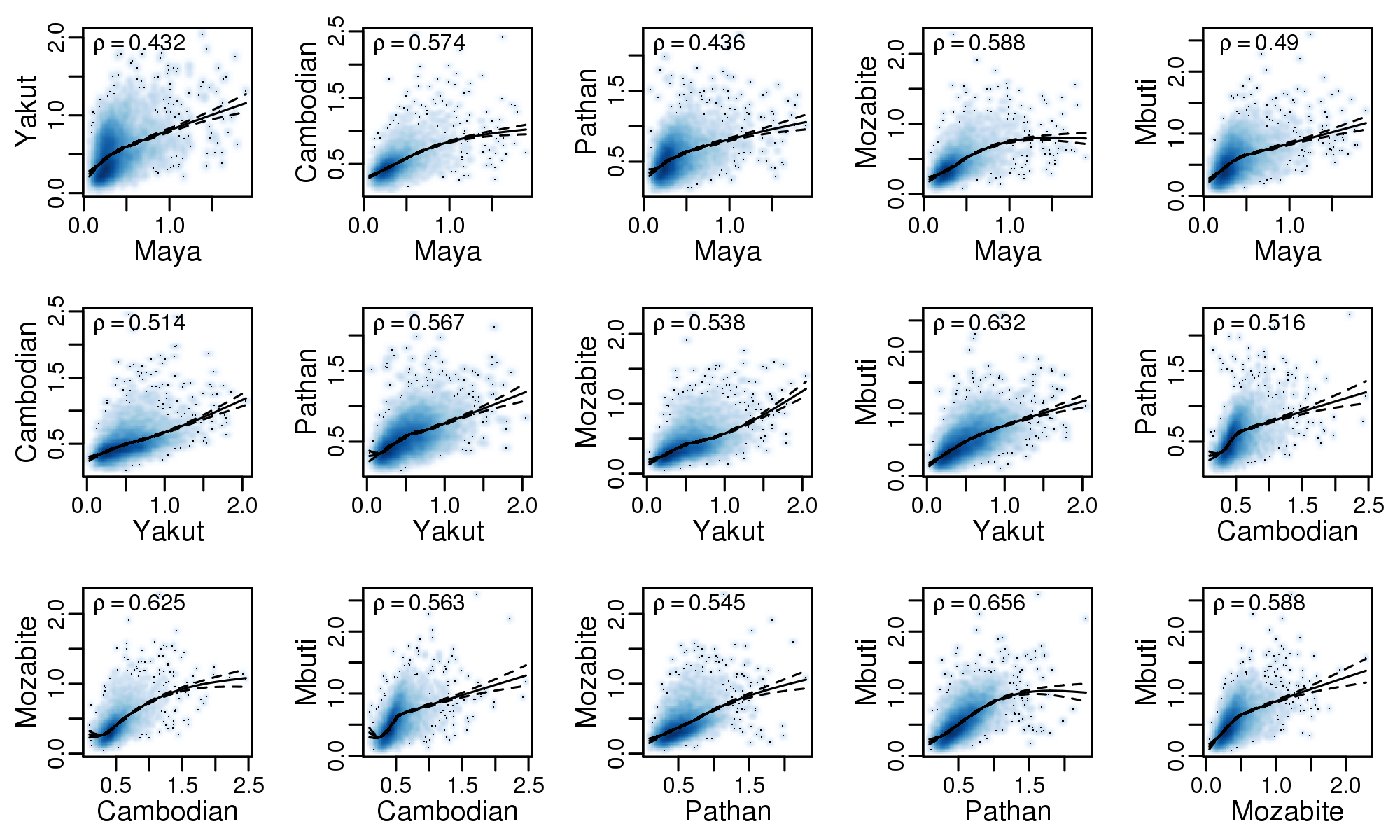

Supplement: Figure S15 — Variability in gene expression across pairwise populations, as measured by the coefficient of variation (cv) which is a measure of gene expression dispersion. Only genes that passed our filters (genes expressed in all individuals, N = 5,334) were included here. (TIF) [file pgen.1004549.s015.tif]

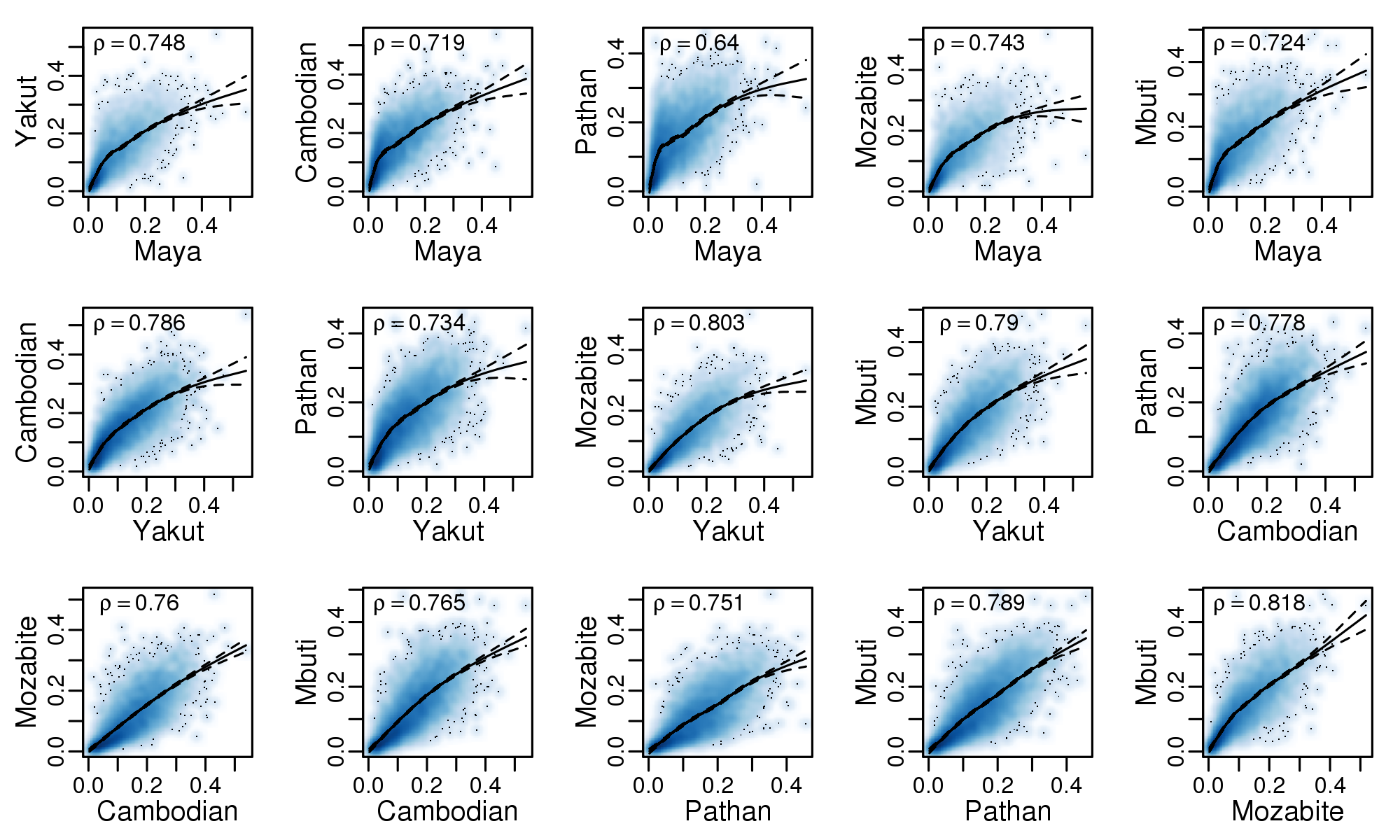

Supplement: Figure S16 — Variability in alternative splicing across pairwise populations, as measured by , i.e. the mean Hellinger distance to the centroid of the relative abundances of alternative splice forms [4]. (TIF) [file pgen.1004549.s016.tif]
